# Bryonia Alba

**Published:** 1885-09

**Authors:** C. S. Lombard

**Affiliations:** Negaunee, Michigan


### CORRESPONDENCE.

NEGAUNEE, Michigan.

*To the Editors of the* CHICAGO MEDICAL JOURNAL AND EXAMINER:

*Messrs. Editors:* One of the most valuable of the drugs known to me is the *Bryonia Alba,* the white bryony of Germany and France. In the human body the sphere of its action is both wide and strikingly manifest. If properly administered, it never fails to produce its curative effects. But the drug has been forgotten. It occupies a scarcely-read page in the National Dispensatory, and barely receives mention in that clever little "EXTRA PHARMACOPŒIA" of Martindale and Westcott.

The white bryony is useful in many pathological conditions. When a patient has a cold involving the lungs, three or four minims of the tincture, largely diluted, may be given with good results, every three hours. In pleurisy, one or two drops may be administered every hour. In bronchitis of the larger tubes three or four minims may be given every three hours, especially if the cough be dry and tight, and the patient complain of painful stitches in the upper portion of the chest. In the bronchial cough of old people, two or three minims given every two to four hours will commonly relieve the symptoms. In all cases the pure tincture should be used.

The drug operates effectively in combination with aconite and gelsemium. If necessary to prescribe two of such medicaments, it is best to give them by alternation.

Bryonia should be tried by a fair test, in order that its real merits may be recognized. It should not be used once timidly, and then, failing to secure the end desired, be laid aside, while a dose of paregoric is substituted. It should not be misjudged because it has achieved popularity with those who call themselves homœopaths. It can be made to appear that its merits are genuine, and its value peculiar to itself, if it be judged without prejudice.

Take, for example, a patient affected with pneumonia, in an early stage of the disease. Let a teaspoonful of the following mixture be given every half hour until the perspiration is freely secreted, and after that once every hour or two:

R.

Bryoniæ Albæ Tincturæ,.................... ♏ x.

Aconiti Radicis Tincturæ,.................♏ xvi.

Aquæ Destillatæ ad,....................... ℥ ii.

M.

In addition to this, give one-half teaspoonful of Horsford's Acid Phosphate in a half glass of cold water, sweetened to suit the taste. Repeat every two hours, gradually decreasing the dose as the temperature shows a tendency to remain near the normal point.

Should it be desired, however, to test the peculiar efficacy of the phosphate in this disease, after having once reduced the temperature from 103° or 104° F. to 99° or 100° F. its use may be suspended for a day and the relapsing fever observed.

If, however, content with having reduced the temperature and with it most of the distressing symptoms, one may continue with the acid and decrease the dose of aconite and bryonia as the case requires. Bryonia has a special action upon, and an affinity for, serous membranes, and if pleurisy complicates the case it may be needed in three minim doses, and as often as every two hours. Conjoined with the foregoing treatment, compresses, large and light, should be thoroughly saturated with vinegar and water, and generously applied to the chest, and if there is much headache, to the forehead, as well. These should be renewed quite often, and by an undisturbing hand. So much for the acute trouble. When active disease has ceased, and it is desired to assist nature in removing pathological products, 1-100 of a grain of corrosive sublimate may be given every three hours.

After having treated a few cases in this way, I do not hesitate in saying that one will become an ardent advocate of the use of byronia.

Since adopting this method of treatment there have been under my care many violent cases, but the record has been a clean one, undefaced by death, and undisturbed by anxiety or alarm.

I have nothing to say to the advocates of Dover's powder, carbonate of ammonia, morphia and quinia, etc., but having tried all these drugs with brilliant success and disastrous failures, I have laid them aside.

Very truly yours,

C. S. LOMBARD, M. D.

